# RNA modifications go viral

**DOI:** 10.1371/journal.ppat.1006188

**Published:** 2017-03-09

**Authors:** Nandan S. Gokhale, Stacy M. Horner

**Affiliations:** 1 Department of Molecular Genetics and Microbiology, Duke University Medical Center, Durham, North Carolina, United States of America; 2 Department of Medicine, Duke University Medical Center, Durham, North Carolina, United States of America; Mount Sinai School of Medicine, UNITED STATES

## Introduction

**Fig 1:**
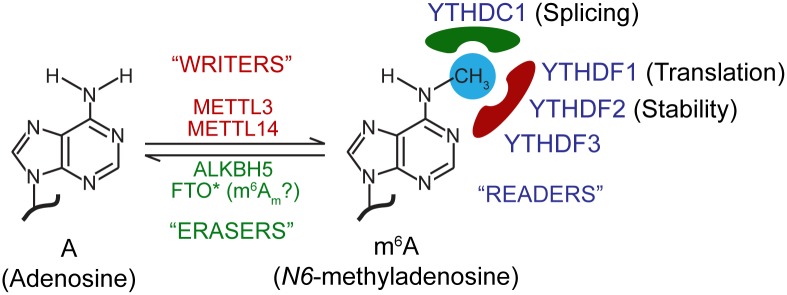
The cellular m^6^A machinery. *N6*-methyladenosine (m^6^A) is a reversible RNA modification that occurs in cellular and viral RNA. The deposition of m^6^A at the consensus motif DRAmCH (where D = G/A/U, R = G > A, and H = U/C/A) is governed by a cellular methyltransferase complex composed of the “writers” METTL3 and METTL14, and other noncatalytic cofactors. m^6^A modification can be reversed by the “erasers” FTO and ALKBH5. *We note that FTO has recently been found to have greater specificity for the m^6^Am modifications present in mRNA cap structures than for m^6^A [34]. “Reader” m^6^A-specific RNA binding proteins, including the cytoplasmic YTHDF1, YTHDF2, YTHDF3, and nuclear YTHDC1 control the function of m^6^A on RNA. YTHDF1 promotes translation of cellular m^6^A-mRNAs, while YTHDF2 targets them for degradation. YTHDC1 regulates the splicing of m^6^A-modified pre-mRNA. The role of m^6^A and the m^6^A machinery in RNA function and biological processes is further reviewed in [2].

Viral life cycles are often coordinated by precise mechanisms that act on their RNA. For example, the microRNA miR-122 interacts with the viral RNA genome of hepatitis C virus (HCV) and is required for HCV replication [1]. In the past year, several groups have reported a new RNA regulatory control to viral infection—the posttranscriptional RNA modification *N6*-methyladenosine (m^6^A). This reversible RNA modification is the most prevalent internal modification of the more than 60 known chemical modifications in eukaryotic RNA. The deposition of m^6^A on RNA is controlled by cellular m^6^A machinery comprising methyltransferase and demethylase enzymes, as well as m^6^A-specific binding proteins (recently reviewed in [2]; Fig 1). By affecting mRNA and noncoding RNA structure, localization, and function, m^6^A plays an important role in many fundamental biological processes [2].

A role for m^6^A in viral infection has been hypothesized since the 1970s, when m^6^A was found on RNA of several viruses [3–7]. Recently, advances in sequencing-based strategies used to profile m^6^A have expanded the known repertoire of viruses with m^6^A in their RNA to include human immunodeficiency virus 1 (HIV-1) and RNA viruses in the family *Flaviviridae*, such as HCV and Zika virus (ZIKV; Table 1) [8–12]. In this article, we will review the emerging role for m^6^A in regulating viral infection.

**Table 1:** List of viruses known to contain m^6^A in their RNA.

Virus	Summary of knowledge	References
**DNA viruses**		
Simian virus 40	Viral late transcripts have ~3 internal m^6^A residues. Blocking m^6^A with cycloleucine impairs nuclear processing and export of late viral mRNAs.	[3, 17, 28]
Adenovirus-2	Viral RNAs contain internal m^6^A. Prior to splicing, viral RNA is modified by m^6^A. m^6^A is retained in the viral mRNA after nuclear export.	[4, 29]
Herpes simplex virus	Viral mRNAs contain internal m^6^A.	[5]
**Retroviruses**		
HIV-1	Viral mRNA and genomes contain m^6^A, which is concentrated at 3’ regions. m^6^A sites at the Rev-response element RNA structure alter nuclear export of viral RNA. m^6^A-binding YTHDF proteins bind to viral RNA, promote viral replication, and may suppress genomic RNA reverse transcription following infection.	[8, 9, 10]
Rous sarcoma virus	Genomic RNA has ~10–15 m^6^A residues per molecule mostly in the 3’ terminal third of the genomic RNA. Blocking m^6^A by cycloleucine reduces formation of the mature, spliced *Env* mRNA.	[6, 13, 14, 19, 21, 22, 26, 27]
Feline leukemia virus	Genomic RNA contains internal m^6^A modification.	[15]
Moloney murine leukemia virus	Genomic RNA contains internal m^6^A modification.	[16]
**(+)-stranded RNA virus**		
HCV	The viral RNA genome has multiple internal m^6^A sites. m^6^A suppresses viral particle production, but does not affect viral RNA replication. YTHDF proteins suppress viral particle production, and relocalize to viral assembly sites around lipid droplets. Mutation of one cluster of m^6^A sites increases viral particle production.	[11]
ZIKV	The viral RNA genome has multiple internal m^6^A sites, with differences in m^6^A modification patterns in 3 strains. m^6^A and YTHDF proteins suppress viral infection.	[11, 12]
Dengue, Yellow fever, and West Nile virus	The viral RNA genome has multiple internal m^6^A sites.	[11]
**(-)-stranded RNA viruses**		
Influenza A virus	Viral mRNAs and genomic RNA segments have internal m^6^A. m^6^A is unequally distributed on viral mRNAs.	[7, 18]

YTHDF, YTH domain family.

## A historical perspective on m^6^A in viral RNA

Early work on RNA modifications in the 1970s often used viral systems to characterize RNA modifications, including the mRNA “cap” structures. Such early studies, which used chromatographic analysis of radiolabeled and enzymatically digested RNA, uncovered a high degree of internal m^6^A modification in cellular mRNA and viral RNAs from the DNA viruses simian virus 40 (SV40), adenovirus-2, and herpes simplex virus 1 [3–5]. m^6^A was also found in the viral genomic RNA of multiple retroviruses and in the mRNA from influenza A virus, a negative-stranded RNA virus [6, 7, 13–16]. Interestingly, all these viruses have a nuclear stage in their life cycle, which led the field to believe that the nucleus was the primary site of m^6^A modification of RNA.

Subsequent experiments mapped the m^6^A sites in viral RNAs, revealing an interesting heterogeneity in viral m^6^A patterns. Rous sarcoma virus genomic RNA contained 10–15 m^6^A modifications per molecule, all localized to the 3’ half of genomic RNA, while m^6^A in SV40 and adenovirus-2 mRNA was near spliced regions [4, 14, 17]. Furthermore, the number of m^6^A residues on individual segments of influenza A virus mRNAs varied greatly between segments [18]. These viral m^6^A-mapping studies revealed a putative consensus motif for m^6^A: GA^m^C and AA^m^C, which was also later confirmed in cellular mRNA [17, 19, 20]. Indeed mutation of GAC to GAU in a cluster of two such motifs in Rous sarcoma virus prevented m^6^A modification at these sites [21, 22]. Modern sequencing techniques to detect m^6^A based on enrichment of m^6^A-modified RNA fragments using an m^6^A-specific antibody (m^6^A-seq), as well as biochemical analyses of the specificity of the m^6^A methyltransferase complex, have validated the early findings on viral RNA. The consensus motif for m^6^A is now known to be DRA^m^CH (where D = G/A/U, R = G > A, and H = U/C/A) [23–25].

Early research on m^6^A in viral infection pointed to the modification regulating viral RNA splicing. The m^6^A-methylation inhibitor cycloleucine reduced splicing of the Rous sarcoma virus *Env* mRNA and impaired the proper nuclear processing and export of SV40 late mRNA [26–28]. Furthermore, m^6^A was proposed to regulate the splicing of adenovirus-2 late transcripts [29]. Indeed, m^6^A has now been to shown to regulate mRNA splicing, highlighting the value of these early viral studies in uncovering m^6^A function [30].

## Recent advances in m^6^A in viral RNA

The recent identification of the cellular m^6^A machinery (see Fig 1) now allows for mechanistic studies on the function of this RNA modification during viral infection [2]. Recently, three groups have found a proviral role for m^6^A in HIV-1 infection [8–10]. Interestingly, these studies found that the function of individual m^6^A sites in HIV-1 RNA can be varied, ranging from regulating HIV-1 RNA nuclear export to enhancing viral gene expression [8, 9]. Furthermore, the m^6^A-binding cytosolic YTH domain family (YTHDF) proteins were found to bind to HIV-1 RNA at m^6^A sites [9, 10] but have varied roles in regulating HIV-1 infection, from promoting viral transcript abundance and translation to suppressing viral reverse transcription [9, 10]. Given that m^6^A regulates splicing during infection by other retroviruses, HIV-1 mRNA splicing may also be affected by m^6^A and by YTHDC1, a nuclear YTH domain containing m^6^A-binding protein involved in cellular mRNA splicing [30]. While this work has uncovered m^6^A and the m^6^A machinery as important regulators of HIV-1 infection, one general limitation of experiments involving the knockdown of the cellular m^6^A machinery is that such depletion could affect the expression of pro- or anti-viral host factors, leading to an indirect effect on viral infection. Experiments involving viruses that contain m^6^A-abrogating mutations will be invaluable in pinpointing the direct role of this modification on viral RNA during infection.

We, and others, have recently found a role for m^6^A in regulating RNA viruses of the *Flaviviridae* family. Using m^6^A-seq, we mapped several regions modified by m^6^A across the RNA genomes of the *Flaviviridae* members HCV, ZIKV, dengue virus, yellow fever virus, and West Nile virus [11]. Concurrently, another group also identified m^6^A in ZIKV RNA [12]. These viral RNAs are the first examples of exclusively cytoplasmic RNA species that contain m^6^A, indicating that the cellular m^6^A methyltransferases may be active in the cytoplasm under some cases. Indeed, the m^6^A methyltransferases are present in the cytoplasm as well as the nucleus [11]. Perhaps they are targeted to viral RNAs by cellular factors yet to be defined that modulate the specificity and localization of the methyltransferase complex. We also tested if m^6^A had any role in *Flaviviridae* infection, and found that m^6^A suppressed the packaging of HCV RNA into infectious viral particles. A conserved cluster of four m^6^A sites in the HCV E1 gene was the primary driver of this phenotype, such that abrogation of m^6^A in this region by mutation altered RNA–protein interactions required for viral assembly [11]. Similar to our work, Lichinchi et al. found that m^6^A also limited ZIKV infection, suggesting that m^6^A negatively regulates *Flaviviridae* infection [11, 12]. Both of these studies mapped the *Flaviviridae* m^6^A sites at a single time point of infection, catching only a snapshot of the overall m^6^A profile on the viral genomes. As current m^6^A-mapping technologies do not allow us to easily determine the m^6^A occupancy of any individual site or whether it occurs on the same viral RNA species, it is likely that these viral genomes will have divergent m^6^A sites and occupancies at different stages of their life cycles. For example, we found that virion-associated RNA had less overall m^6^A than intracellular replicating HCV RNA [11]. By expanding these studies to capture the viral m^6^A sites over a time course of infection or on specific viral RNA species, we could identify new controls governed by m^6^A that regulate specific aspects of viral replication, including viral RNA stability, translation, replication, packaging, or even immune evasion (see below). Furthermore, as m^6^A destabilizes RNA secondary structure [2], it could directly alter *cis*-regulatory structural elements in RNA virus genomes.

The presence of RNA modifications on viral RNAs may prevent detection by host pattern recognition receptors that trigger antiviral innate immunity. Indeed, two studies have shown that internal m^6^A modification of in vitro synthesized RNAs ameliorates innate immune activation by the known RNA-sensing pattern recognition receptors TLR3 and RIG-I [31, 32]. Therefore, m^6^A-modification of the pathogen-associated molecular patterns within viral RNA may be an evolutionary adaptation for immune evasion. Identifying m^6^A modification in viral RNA pathogen-associated molecular patterns during infection will be critical in proving that m^6^A serves as a shield on viral RNA to prevent induction of antiviral signaling pathways.

## Epitranscriptomic changes to host mRNA during viral infection

Viral infection induces broad changes in the host transcriptome and proteome. Therefore, it is not surprising that viral infection can also alter the m^6^A-epitranscriptome in host mRNA. Indeed, both HIV-1 and ZIKV impact the host m^6^A-epitranscriptome with changes to the specific transcripts containing m^6^A and to the overall m^6^A-topology [8, 12]. Specifically, during viral infection, the level of m^6^A increases at the 5′UTR of mRNAs, with a concomitant decrease in m^6^A modification at 3’UTRs. A similar increase in m^6^A at 5’UTRs has been reported in heat shock–related transcripts during heat shock, which promotes the translation of these mRNAs [33]. Interestingly, during viral infection, many m^6^A-altered transcripts are related to viral replication and immune responses [8, 12]. Therefore, m^6^A modification to specific mRNAs could be virally induced to promote infection, or by the host to restrict infection, allowing for an additional layer of gene expression regulation. Future studies on viral- or host-mediated epitranscriptomic changes and identification of the factors that regulate these altered epitranscriptomes will be essential to understanding how viral infection alters host gene expression.

## Conclusions and future perspectives

As important posttranscriptional modulators of RNA function, m^6^A and other RNA modifications likely regulate infection by all classes of viruses. Recent scientific and technological advances have now set the stage for the systematic exploration of many outstanding questions regarding the role of m^6^A during viral infection. Going forward, perturbing the host m^6^A machinery and mutating m^6^A motifs in viral RNAs will be invaluable techniques used to study the function of m^6^A on viral RNA structure, localization, splicing, stability, translation, and immune evasion. Furthermore, understanding viral- or host-induced changes in the cellular m^6^A epitranscriptome will be crucial in understanding gene regulation during viral infection. Indeed, as for many fundamental biological systems, viral infection may prove to be a useful model for understanding how m^6^A affects cellular RNA expression and function. Therefore, we expect that virology and its exciting discoveries will be at the heart of the renaissance of m^6^A and RNA modification research.
